# Improving adherence to guideline recommendations in dementia care through establishing a quality improvement collaborative of agents of change: an interrupted time series study

**DOI:** 10.1186/s43058-020-00073-x

**Published:** 2020-09-24

**Authors:** Kate Laver, Monica Cations, Gorjana Radisic, Lenore de la Perrelle, Richard Woodman, Janna Anneke Fitzgerald, Susan Kurrle, Ian D. Cameron, Craig Whitehead, Jane Thompson, Billingsley Kaambwa, Kate Hayes, Maria Crotty

**Affiliations:** 1 College of Medicine and Public Health, Flinders University, Adelaide, Australia; 2 NHMRC Cognitive Decline Partnership Centre, Sydney, Australia; 3 Griffith Business School, Griffith University, Gold Coast, Australia; 4 Faculty of Medicine and Health, University of Sydney, Sydney, Australia

**Keywords:** Quality improvement, Dementia, Clinical practice guidelines

## Abstract

**Background:**

Non-pharmacological interventions including physical activity programmes, occupational therapy and caregiver education programmes have been shown to lead to better outcomes for people with dementia and their care partners. Yet, there are gaps between what is recommended in guidelines and what happens in practice. The aim of this study was to bring together clinicians working in dementia care and establish a quality improvement collaborative. The aim of the quality improvement collaborative was to increase self-reported guideline adherence to three guideline recommendations.

**Methods:**

Interrupted time series. We recruited health professionals from community, hospital and aged care settings across Australia to join the collaborative. Members of the collaborative participated in a start-up meeting, completed an online learning course with clinical and quality improvement content, formed a quality improvement plan which was reviewed by a team of experts, received feedback following an audit of their current practice and were able to share experiences with their peers. The primary outcome was self-reported adherence to their guideline recommendation of interest which was measured using checklists. Data were collected monthly over a period of 18 months, and the study used an interrupted time series design and multilevel Poisson regression analysis to evaluate changes in self-reported adherence.

**Results:**

A total of 45 health professionals (78% therapists) from different sites joined the collaborative and 28 completed all requirements. Data from 1717 checklists were included in the analyses. Over the duration of the project, there was a significant increase in clinician self-reported adherence to guideline recommendations with a 42.1% immediate increase in adherence (incidence rate ratio = 1.42; 95% confidence interval = 1.08–1.87; *p* = 0.012).

**Conclusion:**

Health professionals working with people with dementia are interested in and willing to join a quality improvement collaborative with the goal of improving non-pharmacological aspects of care. Participation in the collaborative improved the quality of care for people with dementia as measured through self-reported adherence to guideline recommendations. Although there are challenges in implementation of guideline recommendations within dementia care, the quality improvement collaborative method was considered successful. A strength was that it equipped and empowered clinicians to lead improvement activities and allowed for heterogeneity in terms of service and setting.

**Trial registration:**

ACTRN12618000268246

Contributions to the literature

- Quality improvement collaboratives have been shown to improve the quality of care but there are few examples of their use in dementia care.
- Although there are challenges in recruiting and retaining health professionals in aged care, we successfully recruited health care professionals and formed a quality improvement collaborative dedicated to improving non-pharmacological care for people with dementia.
- Self-reported adherence to guideline recommendation increased steadily over the 18 months of the project and improved substantially from baseline levels.

## Background

Dementia is considered a global health priority by the World Health Organization and is a leading cause of death and disability worldwide [1]. In 2015, approximately 47 million people worldwide were living with dementia and numbers are expected to increase in line with an ageing population [2]. Despite an increasing volume of high-quality dementia research, there is limited awareness of effective evidence-based treatments for people with dementia [3]. As such, the care of people with dementia has been adversely affected by therapeutic nihilism, where there is scepticism regarding the benefit of treatments [4, 5]. The development of clinical practice guidelines for dementia has aimed to increase awareness of the evidence and subsequently improve the quality and consistency of dementia care [6, 7]. The first clinical guidelines for dementia to be endorsed by Australia’s National Health and Medical Research Council were published in 2016. The guidelines were supported by a number of different professional associations and the country’s peak consumer organisation. The guidelines provide recommendations relating to the assessment and care of people with dementia in community, hospital and residential settings and contain evidence-based recommendations and practice points. However, the production of clinical practice guidelines alone may not have a subsequent impact on practice. As described by Glasziou and colleagues [8], health professionals in the field must be aware of and accept guideline recommendations. They must believe that guideline recommendations are applicable to their workplace and they must be able to implement recommendations within existing resources. Furthermore, they must disrupt the status quo and take action to implement changes.

Governments worldwide are grappling with how to provide quality and cost-effective care for an increasing number of people with dementia [2]. It is critical to trial and evaluate interventions which can reduce the gap between evidence (as detailed in guideline recommendations) and practice. Within the field of dementia care, there are specific challenges related to the implementation of guideline recommendations. There are usually several health professionals involved in the person’s health and aged care (such as the general practitioner, geriatrician, nursing, allied health and care workers), and integration of care is limited in most regions [9]. In addition, recruiting and retaining health professionals is difficult, impacting continuity of care [10]. Enablers that promote successful implementation in dementia care include providing staff with knowledge and training in implementation, coaching, group and individual learning activities, dedicated time to implement strategies, organisational support and a clear understanding of the expected behaviour change [11, 12].

Strategies found to enable guideline implementation have previously been packaged as quality improvement collaboratives and have yielded promising success across health care fields. Quality improvement collaboratives have been used in other fields of healthcare with success [13]. Collaboratives generally involve (1) a focus on a specific topic, (2) clinicians from multiple sites, (3) a team of clinical and quality improvement experts available, (4) structured activities to promote learning, and (5) a model for quality improvement that tracks progress against measurable aims [14]. Wells and colleagues conducted a systematic review including 64 studies of quality improvement collaboratives and reported that, overall, collaboratives were associated with improvements in both process and clinical outcomes [13].

There are few examples of quality improvement collaboratives within the fields of gerontology and geriatrics [13] although studies are underway [15]. The field of dementia care is unique and consideration must be given as to how to adapt collaboratives accordingly. It is important that quality improvement collaboratives established within dementia care settings are flexible enough to accommodate participants from different settings and professional backgrounds, and who work within different models of funding. It is also important in a country like Australia, which has a low population density, to take advantage of technologies such as videoconferencing, which allow for meetings of the quality improvement collaborative without the time and cost associated with travel. Furthermore, with different time zones and schedules, it can be difficult to identify mutually convenient times for the collaborative to meet and learn. The flexibility of e-learning activities is therefore valued by health professionals [16].

The aim of this study was to determine whether the establishment of a national quality improvement collaborative of health professionals could increase self-reported guideline adherence to recommendations in dementia care (and by implication, an improved quality of care for people with dementia). Members of the national quality improvement collaborative were described as being ‘agents of change’. Specifically, members within the collaborative were interested in adherence to one of the three following guideline recommendations [6]. These recommendations were selected for the quality improvement collaborative as they are priorities for people with dementia and their care partners [17], but adherence is known to be poor [18].

1. People with dementia living in the community should be offered occupational therapy (reflecting evidence-based programmes)
2. People with dementia should be strongly encouraged to exercise
3. Care partners and family of people with dementia should have access to programmes that provide respite and support to optimise their ability to provide care for the person with dementia.

This paper addresses the following research questions:

1. Can the establishment of a national quality improvement collaborative of health professionals increase self-reported adherence to three recommendations from clinical practice guidelines for dementia? If so, are increases sustained over the following months?
2. What is the impact of the quality improvement collaborative on experiences and outcomes for people with dementia and their care partners?
3. Do the health professional members of the quality improvement collaborative value participation?

## Methods

**Fig. 1:**
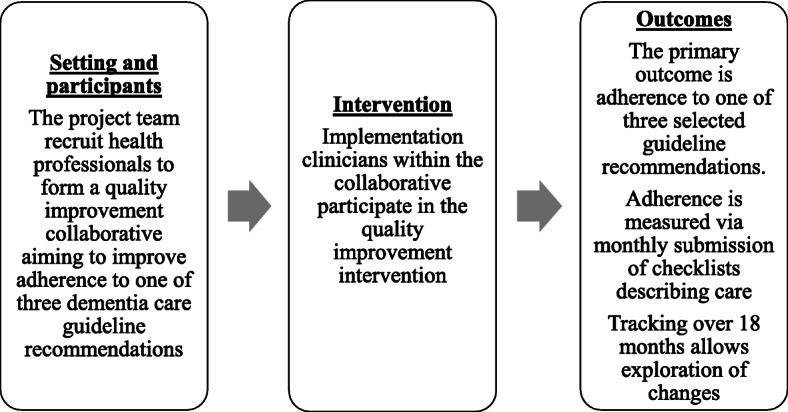
Project overview

The study protocol for this implementation research study was previously published and presents a detailed description of methods [19]. This paper describes the outcomes for research questions 1 and 3 in the protocol paper. Research questions 2 and 4–7 were answered through a comprehensive process evaluation which involved interviews with collaborative members throughout the process and economic analysis. The process evaluation generated a large volume of interesting data which will be reported separately. An overview of the research design is presented in Fig. 1. Ethical approval was received from the Southern Adelaide Clinical Human Research Ethics (number 62.17) and governance approval was received at all participating sites. The study used an interrupted time series design to assess self-reported guideline adherence (the primary outcome) with monthly data collection over a period of 18 months during 2018 and 2019. Interrupted time series are considered one of the strongest experimental designs where randomisation is not appropriate [20]. The design is particularly popular in translational research studies where the aim is to offer evidence-based interventions and it is not ethical to withhold evidence-based intervention to a control group.

### Setting and participants

We recruited health professionals (referred to hereafter as ‘implementation clinicians’) from health and aged care organisations and sites (‘implementation sites’) across Australia. Eligible health professionals were registered with the appropriate registration board, regularly working with people with dementia and/or their care partners, with influence within their workplace (and possibly leadership responsibilities) and with clinical responsibilities. We advertised via professional associations, aged care organisations, peak bodies (such as Leading Age Services Australia) and health services. Interested health professionals were required to submit a written ‘expression of interest’ along with a signed statement from their manager or supervisor demonstrating support for their participation. Interested health professionals were invited to join the collaborative if they worked in a range of different settings including general practice, community care, memory clinics, residential care, private practice or hospital services and rural and metropolitan areas. Upon applying, the health professional had to state which guideline recommendation (out of the three recommendations which were the focus of this project) they wanted to address to improve adherence.

### Intervention: implementation strategies

The (implementation) intervention involved identifying and recruiting health professionals to form a national quality improvement collaborative. The intervention elements of the quality improvement collaborative included the following:

- A face-to-face start-up meeting which involved introductions to the research team and other members of the collaborative. This was followed by presentations about evidence for dementia care interventions, dementia guidelines and quality improvement. The meeting was held in person, was facilitated by the project team and invited speakers and lasted 3 h.
- An online, eight module, evidence-based education package (hosted on the OpenLearning platform) comprising written materials, videos, learning activities and chat function. The first modules summarised the latest evidence in dementia care related to the guideline recommendations in scope for this project. The latter modules provided a brief overview of theories in knowledge translation and quality improvement and then practical applications. Clinicians worked through stakeholder analysis, identifying barriers and enablers and then designing their own implementation plan using a plan-do-study-act (PDSA) model. The project team offered clinicians individual support (over phone, videoconference or email) to develop their plan if needed. Each completed plan was reviewed with feedback provided by a clinical expert, implementation expert and person with lived experience of dementia (as a person with dementia or as a carer). We had initially planned to offer seven modules (as described in our protocol) but realised that it was necessary to add an eighth module following implementation of the PDSA cycles which facilitated reflection and topics about persistence and sustainability.
- A single instance of feedback on performance against audit criteria. These were staggered at two time points in order for the team to manage the workload (as per Figure A in supplementary material which provides a timeline for the project). We provided each clinician with feedback on their ‘submitted checklists’ (described below) to date; their level of self-reported adherence to the guidelines as per the scoring criteria explained below and some individualised feedback as to why they may not have been consistently adhering to the recommendations (e.g. they were not routinely providing a written care plan).
- Access as required to ongoing clinical and quality improvement expertise within the project team via phone, email or videoconference.
- Regular project incentives (as per the timeline which is presented as Figure A in the supplementary material online) including a welcome pack, book on evidence-based health care and motivational gift pack. Upon joining the collaborative, clinicians were informed that they would be able to access a stipend of up to AU$1000 to cover the costs of sharing the results of their work at a self-organised presentation or an external meeting or conference. The stipend could be used for travel and/or registration costs.
- Access to webinars designed to meet the learning needs of the clinicians which were exclusively available to members of the collaborative.
- Monthly videoconference meetings facilitated by the project team which provided an opportunity to discuss progress and challenges and discuss their work with their peers.

These strategies supported clinicians through the quality improvement process. They identified gaps in practice, mapped key stakeholders and brainstormed barriers and enablers to change. They used this information to create change via iterative PDSA cycle s[21]. A timeline detailing the contents of intervention and timing is provided in supplementary material online (Figure A). The intervention was specifically designed to allow for heterogeneity in terms of clinicians, client groups and sites and to recognise the expertise and knowledge of context and setting that individual clinicians possess. Based on known enablers and barriers in dementia care, we identified key attributes necessary for a quality improvement collaborative to be feasible. These factors are detailed in supplementary material (Figure B). Briefly, key barriers included limited, time, support and knowledge in how to enact quality improvement activities in practice. Clinicians were not paid to be involved in the collaborative and participation required an investment of their time.

### Outcomes

The primary outcome for this implementation research study was self-reported adherence to the selected guideline recommendation. In consultation with clinical and lived experience experts, we transformed three existing clinical practice guideline recommendations for dementia into criteria that could be checked and audited. The recommendations were sourced from the Australian Clinical Practice Guidelines and Principles of Care for People with Dementia [6] and related to evidence-based occupational therapy, encouragement to exercise and care partner support as described previously. Scoring criteria are presented in Table 1.

**Table 1:** Criteria developed to measure self-reported guideline adherence

Guideline adherence	
Exercise guideline adherence	Full adherence when: a. Clinician checklist explicitly references a discussion about current physical activity levels b. Specific needs and barriers to physical activity are identified c. Treatments/strategies recommended are clinically indicated based on needs/barriers d. A written treatment plan for physical activity or exercise is provided to the person with dementia
Occupational therapy guideline adherence	Full adherence when: a. Home environment assessment has occurred (where applicable) b. Clinician checklist explicitly references identification of primary concern/s of a person with dementia and care partner c. A written treatment plan to address the needs of a person with dementia and care partner or give specific advice about suitable activities (that are tailored, of interest, and match capabilities) is provided
Care partner support guideline adherence	Full adherence when: a. Clinician checklist explicitly references that the needs of the care partner have been discussed during the consultation b. Clinician checklist explicitly references clinically indicated provision of information about programmes providing respite for the care partner and/or other care partner support services c. A written treatment plan detailing key care partner concerns and strategies to manage these is provided

In the absence of common datasets or standardised reporting mechanisms, we evaluated adherence to guideline recommendations through reporting checklists. An example of a checklist is presented in supplementary material online (Figure C). Implementation clinicians completed a checklist following each consultation they completed with a client with dementia. We asked clinicians to submit checklists for up to ten clients per month (representing their first ten consecutive consultations for the month with people with dementia). We were mindful of capturing data which were representative of their practice but not overly burdensome to collect and therefore a barrier to participation in the collaborative. Checklists were scored independently by two people with a 0 representing non or partial adherence or 1 representing full adherence. In cases of disagreement, a third person was sought to make a decision. This scoring method differed slightly from our protocol where we initially planned a three-point scale (− 1, 0 or 1). We changed our approach as it became clear that there was little use in differentiating between ‘inadequate’ or ‘partial’ adherence. To address research question 2 of the study (i.e. outcomes for people with dementia and their carers), we contacted a random sample of the implementation clinician’s clients with dementia (or their care partners) who received the reported consultation by phone to verify the clinician’s account of the consultation. The aim of the phone interview was to obtain the perspectives of the person with dementia and their care partner about the consultation and adherence to the criteria. We asked people with dementia and their care partner a number of questions about their satisfaction with care, quality of life and level of burden reported by care partners. Health professionals in the collaborative provided information about their demographics through a survey which they completed upon enrolment in the collaborative.

### Analysis

Demographic and checklist data were entered into Excel and then exported into STATA (StataCorp, USA, version 16.0) for analysis. Descriptive analysis was performed using, means ± standard deviation, medians (inter-quartile range) or frequencies (percentages) as appropriate. As per the criteria set out in Table 1, consultations (as described within clinician checklists) were scored by an independent assessor as being either in full adherence with the associated guideline recommendation (score = 1) or not in full adherence (score = 0). We used an interrupted time series analysis approach to assess the changes in self-reported clinician adherence after December 2018 when the key elements of the intervention were completed. For this, a multilevel Poisson regression analysis was used. A Poisson distribution was chosen in order to assess the relative rate of change in adherence. A multilevel model was used in order to account for the repeated measures on clinicians across time. The model included fixed effects for month, period (before versus after Dec 2018) and a month X period interaction. A random intercept was also included for the participant to account for the correlation in the data. The fixed effect term for ‘period’ allowed us to assess the immediate change in the rates of clinician adherence in December 2018, i.e. the ‘level’ change. The ‘month’ term allowed us to assess the relative increase in adherence prior to December 2018, i.e. the month-to-month increase. The ‘month X period’ interaction term allowed us to assess whether the slope after December 2018 changed significantly compared to the slope before December 2018. A 2-sided type 1 error rate of alpha = 0.05 was used for significance testing.

## Results

**Fig. 2:**
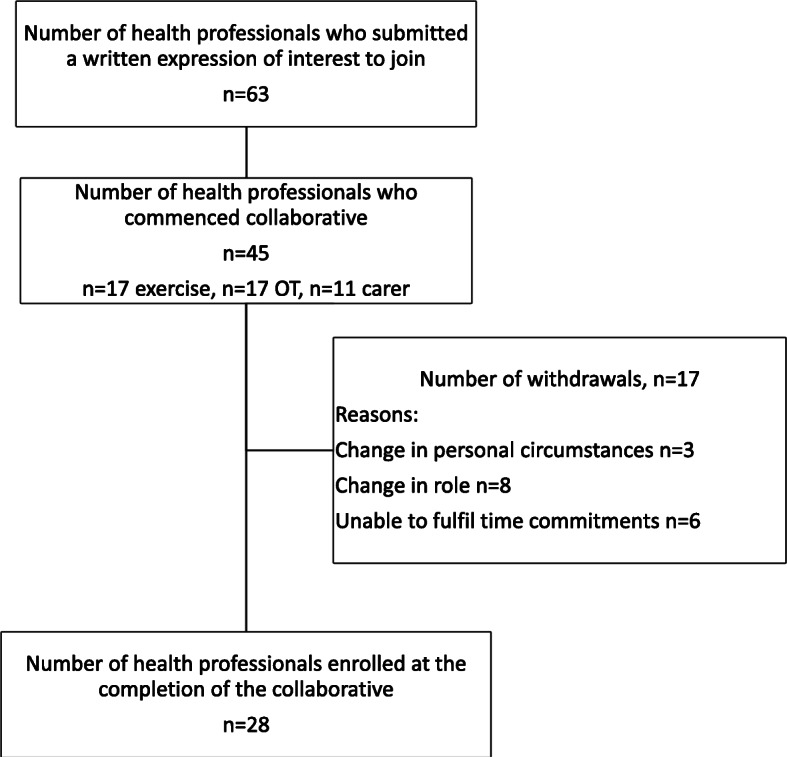
Overview of recruitment and withdrawals

A total of 63 health professionals submitted written expressions of interest to participate in the collaborative. A total of 45 health professionals met all requirements and commenced participation. Over the course of the project (which included several months to obtain research governance approvals at all participating sites as well as 18 months of data collection), there were several withdrawals and a total of 28 (62%) clinicians remained in the collaborative at the conclusion of the project. Recruitment and withdrawals are presented in Fig. 2. Reasons for withdrawal included a change in role, change in personal circumstance or unable to fulfil the time commitment.

The 45 implementation clinicians who were initially enrolled in the project represented all states and territories of Australia and included seven clinicians (16%) who worked in regional or remote areas. Most clinicians (*n* = 40, 89%) were female and worked on average 0.8 full-time equivalent (FTE) (range 0.2 FTE to 1.0 FTE) which equates to approximately 30 h per week. Characteristics of implementation clinicians are presented in Table 2.

**Table 2:** Characteristics of implementation clinicians

Characteristic	N (out of 45), %
Gender	
Male	5 (11%)
Female	40 (89%)
Professional background	
Occupational therapist	19 (42%)
Physiotherapist	16 (36%)
Clinical nurse consultant	3 (7%)
Medical practitioner	2 (4%)
Health service manager	2 (4%)
Social worker	2 (4%)
Dietitian	1 (2%)
Organisational type	
Public	23 (51%)
Private	7 (16%)
Not-for-profit	15 (33%)
Setting	
Community care	24 (53%)
Inpatient (acute, subacute)	6 (13%)
Residential care	8 (18%)
Mixed caseload/other	7 (16%)

### Participation in the quality improvement collaborative and achievement of key milestones

The median amount of time spent participating in the online learning modules was 40 h. All 28 clinicians who completed the collaborative submitted their quality improvement plan for review.

### Self-reported guideline adherence and sustainability

Over the 18-month data collection period, a total of 1717 checklists were submitted (average 95 per month). The number of checklists submitted varied each month (range 55–171) due to changes in workload and the types of clients of the service that month, staff taking leave or changing duties during that month (including covering for other staff on leave at their site).

As seen in Table 3, there was a statistically significant level of change in self-reported clinician adherence to guideline recommendations in December 2018 with an estimated 42.1% immediate increase in adherence (incidence rate ratio (IRR) = 1.42; 95% CI = 1.08–1.87; *p* = 0.012). Prior to December 2018, the relative increase in adherence from one month to the next was approximately 5.3 % (IRR = 1.05, 95% CI = 0.97, 1.14; *p* = 0.225). After December 2018, the relative increase remained the same at approximately 5.3% (IRR for month X period interaction = 0.99; 95% CI = 0.92, 1.09). Overall, the estimated adherence increased from 24.4% in June 2018 to 82.7% in November 2019.

**Table 3:** Immediate (level) and relative (slope) changes in adherence following the introduction of the adherence guidelines

	IRR	95% confidence interval		***p*** value
Period (level change at Dec 2018)	1.421	1.080	1.869	0.012
Month (slope prior to Dec 2018)	1.053	0.969	1.144	0.225
Month X period (additional increase in slope for month after Dec 2018)	0.999	0.917	1.090	0.990

Using a multilevel Poisson regression model with fixed effects for month, period (before versus after Dec 2018) and a month X period interaction. A random intercept was included for the participant

*IRR* incidence rate ratio from Poisson regression model

**Fig. 3:**
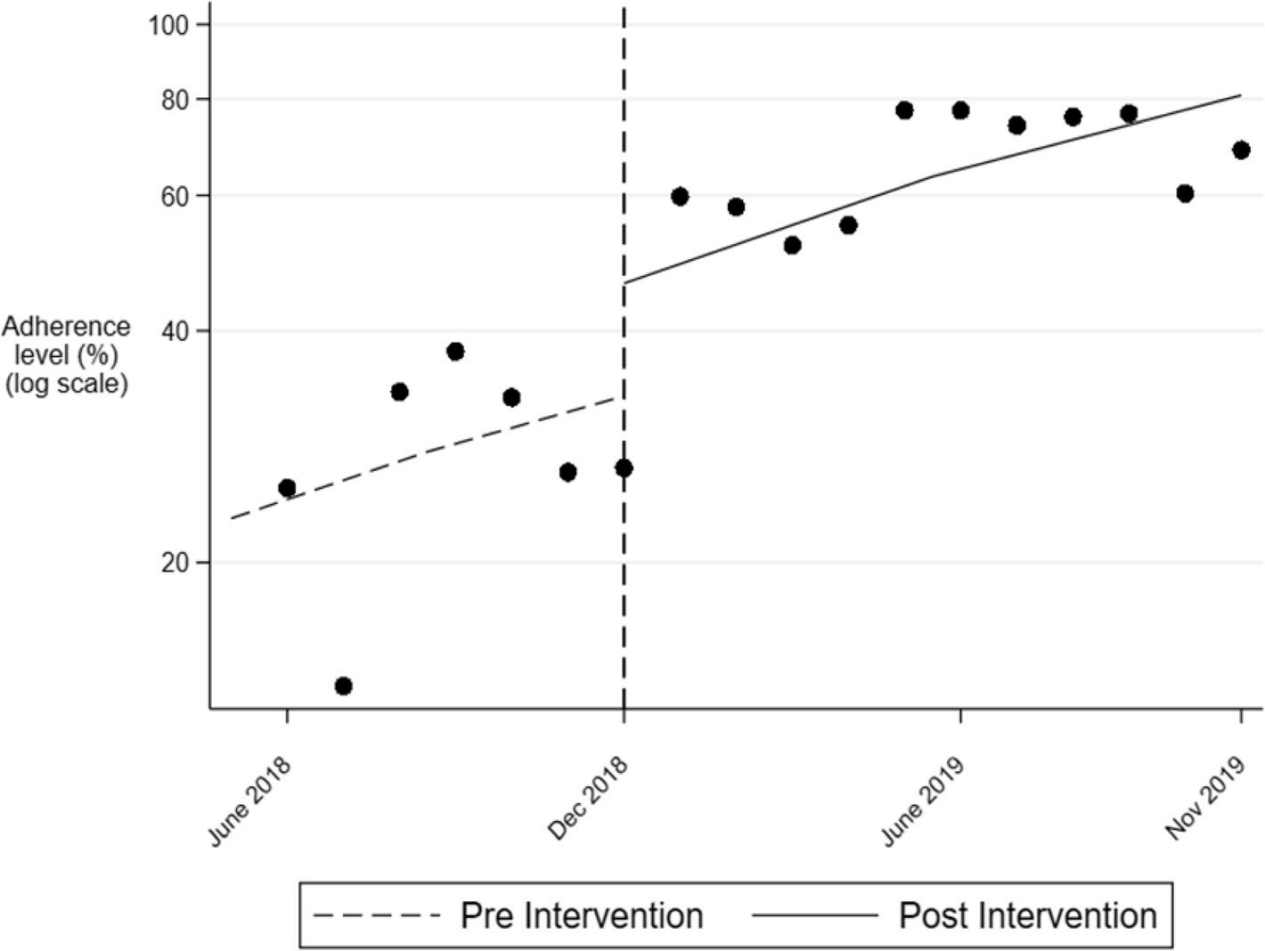
Observed and estimated values of adherence during the study period

The observed and estimated adherence levels are plotted for the two time periods in Fig. 3.

### Outcomes for people with dementia and their care partners

As per our second research question, we had hoped to gain the perspectives of people with dementia and their care partners about the content of consultations, their satisfaction with care and their personal outcomes. However, we were not successful in obtaining these data. There were very few occasions where clinicians gained consent from the person with dementia and their care partner to be contacted. Therefore, we were unable to contact the person with dementia and their care partner to seek their perspectives. Potential reasons for our failure to obtain these data are discussed below (see the ‘Discussion’ section). Clinicians reported that they felt anxious about gaining their client’s consent to participate in a formal research project, that they were confused by the process, that it added extra time burden to consultations and that they did not think that their clients would want to be bothered with a phone call. We chose not to persist with reminders about this component of data collection as we wanted to ensure that we supported implementation clinician participation and engagement and avoided complexity and possible withdrawals. When it was possible to contact people with dementia and their care partners, all provided positive descriptions of their consultations. However, they tended to have difficulty remembering the exact content of consultations. It should be noted that clients would see the implementation clinician a number of times and it was difficult for them to recall the nature of a consultation completed on a particular date.

### Satisfaction with participation

Upon completion of the project, implementation clinicians were asked to complete a survey describing their perspectives of participation. A total of 17 (of 28 completers) clinicians completed the survey, and results are presented in Table 4. Overall, clinicians reported that their quality improvement activities were successful and that the quality improvement skills learnt within the project were advantageous in their workplace.

**Table 4:** Clinician satisfaction with participation (*n* = 17 completed)

Statement	Percent agreeing
Extent to which aims were achieved	
Not at all	6%
Somewhat	24%
In the middle	35%
Mostly	29%
Completely	6%
Extent to which outcomes improved using the selected measures	
Not at all	6%
Somewhat	24%
In the middle	47%
Mostly	18%
Completely	6%
Success implementing quality improvement during the project period	
Not at all	0%
Somewhat	35%
In the middle	24%
Mostly	29%
Completely	6%
Improvements associated with the project were beneficial to	
Myself	71%
Other staff within the organisation	41%
People with dementia	59%
Informal care partners of people with dementia	59%
Other	29%
Frequency of using quality improvement skills learnt within the project	
Never	0%
Occasionally	47%
Often	29%
All the time	24%

## Discussion

This study answered our research questions as to whether the establishment of a quality improvement collaborative could increase self-reported adherence to guideline recommendations, whether adherence was sustained and whether members of the collaborative valued participation.

We found that there was interest amongst clinicians working in dementia care in participating in a quality improvement collaborative focussed on improving non-pharmacological aspects of care. Clinicians were not paid to be involved in the collaborative and participation required an investment of their time. Our successful establishment of the collaborative demonstrates that there are clinicians with an interest in quality improvement activities and a commitment to improve the quality of dementia care. We found a substantial increase in self-reported adherence to guideline recommendations over the duration of the project. Improvements in adherence were relatively steady over time but with a larger increase in adherence after the key elements of the intervention were introduced from December 2018; these were completion of online learning modules, submission of quality improvement plan and feedback following audit of current practice.

While this preliminary work has yet to examine the impact of our quality improvement collaborative on client outcomes, it is plausible that improvements in care quality would in turn result in improved outcomes for clients with dementia. The three guideline recommendations of interest within this project are supported by evidence from multiple randomised controlled trials [6]. There is good evidence that occupational therapy can delay functional decline in activities of daily living and improve quality of life [22]. Increased physical activity is associated with improved mobility and function in people with dementia [23], and there are many research studies which demonstrate the efficacy of caregiver support programmes [24].

The findings of this study are consistent with those from other quality improvement collaboratives which have improved the performance of process outcomes [13]. There have been few examples of quality improvement collaboratives within dementia care settings, but this study suggests that this knowledge translation strategy may be effective in that setting. Systematic reviews suggest that knowledge translation activities are often associated with modest, albeit important, improvements in care [25]. The extent of improvements in self-reported guideline adherence was beyond those anticipated by the research team. Our findings also suggest that transforming guideline recommendations into criteria which can be audited may be beneficial for health professionals to assist with operationalising changes in practice and self-reflection.

The positive effects of the intervention on guideline adherence suggest that this model should be adopted in practice. The benefits need to be considered relative to costs of staff and administration, and we recommend that future quality collaboratives in the field seek to recruit larger numbers of participants to ensure they are worthwhile. Most workplaces require staff to be involved in quality improvement yet offer little support or training in how to do this likely resulting in ineffective or insignificant improvements. Supporting staff to participate in external quality improvement builds skills, capacity and network in staff and should be supported by managerial staff. Withdrawals from the quality improvement collaborative were mostly due to change in role or circumstances (e.g. maternity leave). Retention of staff in aged care organisations is challenging, and we suggest that future quality improvement collaboratives take place over 6 months rather than 18 months. In this study, we required 18 months to ensure the interrupted time series design was adequately powered.

We considered the quality improvement collaborative to be effective in increasing self-reported adherence to guideline recommendations; however, there are a number of limitations which should be acknowledged. Defining and measuring primary outcomes is challenging in translational research involving non-pharmacological interventions. Guideline recommendations describe processes of care which are difficult to quantify and not routinely captured. The lack of consistent and accessible data across sites meant that we developed our own process of measuring outcomes and resulted in our use of checklists which were not validated. These self-reported data were susceptible to bias as are other methods used to measure quality of care such as case note audits and being observed or audio recorded during consultations. We had to design our own checklists as no existing tools were suitable to measure the processes of interest. We had not anticipated that completion and submission of the checklists alone would lead to changes in practice but our results suggest that this may have contributed as there were increases in adherence from the commencement of data collection. Clinicians reported that reflection about practice helped identify the gap and the need for changes in practice in some cases. Therefore, we must consider that some of the improvements in self-reported adherence can be related to checklist completion rather than the establishment of the quality improvement collaborative.

We were unable to answer our second research question regarding the impact of the collaborative on improving satisfaction with care or patient-centred outcomes for people with dementia and care partners. We were unable to successfully obtain data to determine whether increased guideline adherence resulted in improved satisfaction with care or enhanced outcomes for clients of the service as we had great difficulty contacting the clients of our implementation clinicians. We received feedback from implementation clinicians that they did not feel comfortable gaining formal consent from their clients in a research capacity. Many of our clinicians had little previous experience participating in research projects. Gaining consent added to the duration of the consultation, and many of our clinicians reported being time poor (which was also a barrier to best practice and quality improvement).

While interrupted time series studies are highly appropriate in translational research, they do not involve a control group meaning that we must be somewhat guarded when drawing conclusions about the effectiveness of the intervention. The lack of a control group means that the generalisability of the findings is unknown.

There are an increasing number of knowledge translation activities occurring in the field of dementia care and aged care. This is one of the first examples of a quality improvement collaborative and builds knowledge and understanding about what works in this clinical area. We included health professionals from across Australia and had limited dropouts considering the duration of the project (18 months) and the need to obtain research governance approvals at each site. The total number of participants recruited was conducive to working together as one collaborative (dedicated to three recommendations). A larger number of participants would have necessitated breaking into smaller groups. If shown to be effective in pilot studies, quality improvement collaboratives should aim to be larger in scale so that they are able to maximise impact and benefits for people with dementia and their care partners relative to investment. Collaboratives that can be initiated and independently sustained by a group of clinicians (without external support) can reduce costs. However, a clinician-led collaborative would need to consider how it would offer access to advice, coaching and credibility; research establishing how best to achieve this is a worthwhile direction for future research.

## Conclusions

In conclusion, the results of our study suggest that there are motivated health professionals who want to improve the quality of care for people with dementia and care partners and can do so when provided with skill development in quality improvement, support and structure in the form of a quality improvement collaborative. A quality collaborative can support health professionals even when they are working in different contexts in dementia health and aged care organisations.

## Supplementary information

**Additional file 1:.** Figure A: Description and timing of the elements of the intervention. Figure B: Practical design considerations for a quality improvement collaborative in dementia care in Australia. Figure C: Example of completed reporting checklist (example used for the occupational therapy recommendation. Other versions used for exercise and carer support).

## Data Availability

The datasets generated and/or analysed during the current study are not publicly available due to privacy agreements but are available in a decoded and protected form from the corresponding author on reasonable request.

## Abbreviations

IRR: Incidence rate ratio
CI: Confidence interval
FTE: Full-time equivalent
