# Supplementary material for: Improving adherence to guideline recommendations in dementia care through establishing a quality improvement collaborative of agents of change: an interrupted time series study
Source: Implement Sci Commun. 2020 Sep 24;1:80. doi: 10.1186/s43058-020-00073-x (PMC7513321; doi:10.1186/s43058-020-00073-x)

*Supplementary Material*

*Figure A: Description and timing of the elements of the intervention*


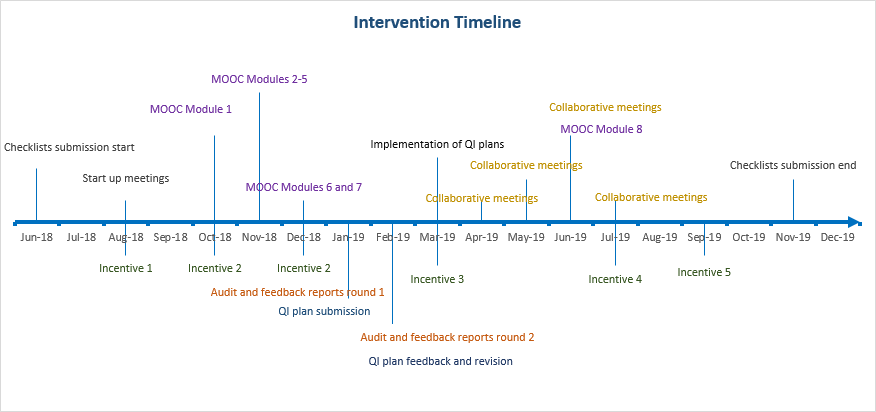


*Abbreviations: MOOC: Massive Open Online Course; QI: Quality Improvement*

List of Incentives

1. Welcome gift pack (tote bag, coffee cup, lanyard, pen with project logos)

2. Webinars provided by expert (topics: Leadership, understanding changed behaviours in dementia)

3. Book: ‘How to implement evidence based healthcare’ by Trish Greenhalgh

4. Motivation gift pack (pen, note pad, smarties, paper clips, rubber bands, inspirational quote)

5. Box of Chocolates

*Figure B:* *Practical design considerations for a quality improvement collaborative in dementia care in Australia*

| **Issue identified** | **Design feature** |
| --- | --- |
| Clinicians need dedicated time to implement strategies and support from their organization | - We recruited clinicians who had leadership responsibilities and were therefore expected to lead quality improvement activities within their role - Clinicians joining the collaborative were required to provide a letter signed by their manager which stated that they would be allocated time to participate and conduct quality improvement |
| Few clinicians would have knowledge or training in quality improvement theory or methods | - Online learning modules involving an overview of theory and practical application and methods of quality improvement. - Individual support from project team available as required (phone or email) |
| Clinicians value a mix of group and individual learning activities and skills based education. | - Group activities (start up meeting, online chat within the modules, allocation to small workgroups, webinars, teleconferences) and individual activities (self paced learning activities throughout the learning modules). A focus on practical applications and case studies. |
| Behaviour change is more likely when expectations are clear | - Clear targets established and measured by the clinicians using the PDSA methodology |
| Clinicians are time poor, may lack control over their daily schedule and have little access to more funds or resources to invest in quality improvement activities | - Clear expectation that quality improvement activities were completed with existing funds and resources within the organization - Flexibility in learning schedule – able to complete the learning modules and quality improvement cycles over months according to own capacity |
| Clinicians work in varied settings, have varied roles and may work in isolated settings (eg sole physio at a residential care facility) or in remote areas | - Emphasis on adapting guideline recommendations to suit individuals own setting. - All activities (except the start-up meeting) held using information and communication technologies. |
| Dementia care and allied health intervention traditionally considered an area to be lacking in evidence | - QIC established following recent launch of Australian clinical practice guidelines. Project team comprising research, clinical and consumer experts providing credibility. |

*Figure C: Example of completed reporting checklist (example used for the occupational therapy recommendation. Other versions used for exercise and carer support).*


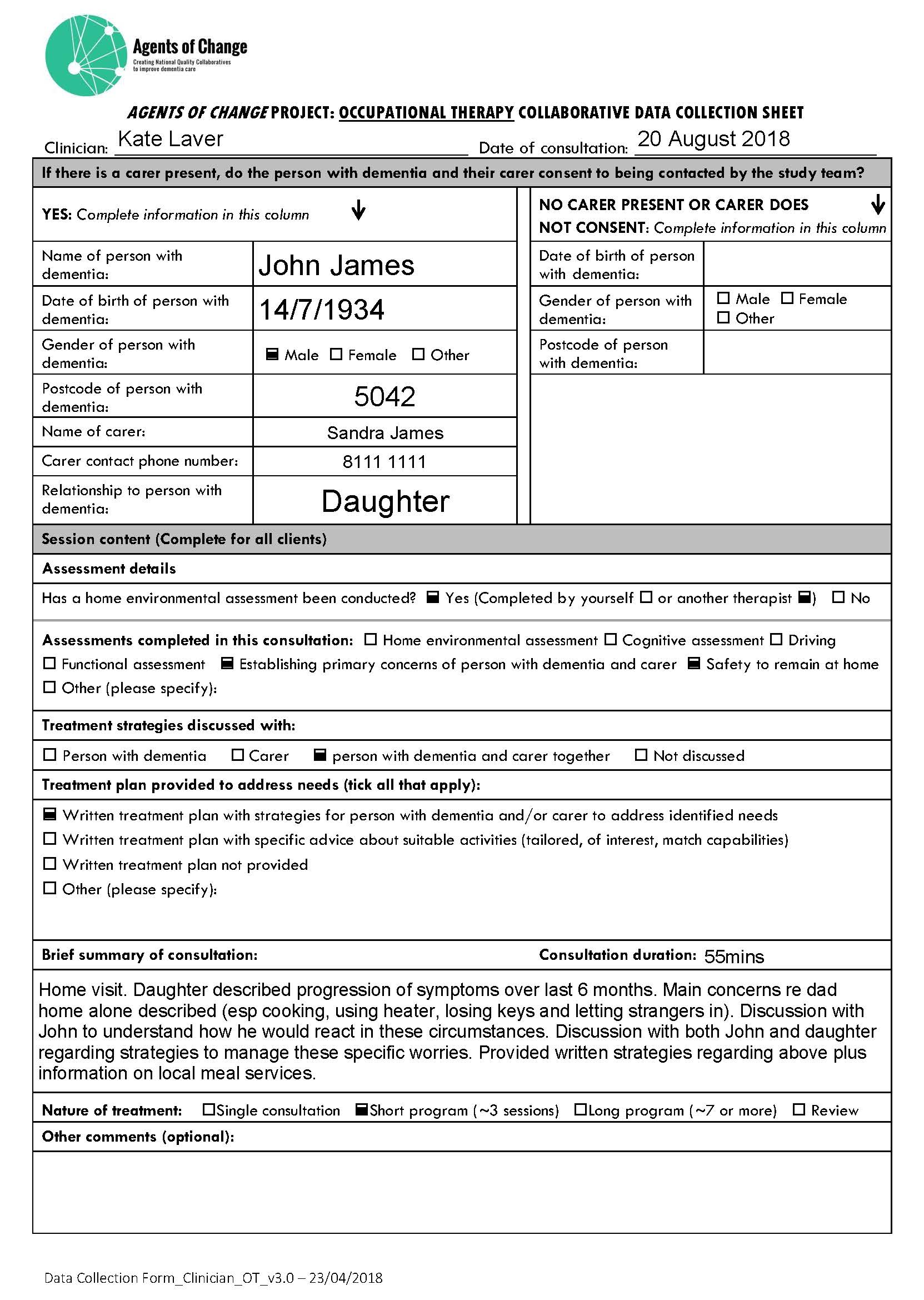

Supplement: Supplementary file 1 — Additional file 1:. Figure A: Description and timing of the elements of the intervention. Figure B: Practical design considerations for a quality improvement collaborative in dementia care in Australia. Figure C: Example of completed reporting checklist (example used for the occupational therapy recommendation. Other versions used for exercise and carer support). [file 43058_2020_73_MOESM1_ESM.docx]
